# The impact of probiotics on glycemic control during the first 3 months after sleeve gastrectomy: a prospective placebo‑controlled study

**DOI:** 10.20452/wiitm.2025.17968

**Published:** 2025-07-04

**Authors:** Dominika Mysiorska, Natalia Mazek, Julia Młyńska, Ewelina Sosnowska‑Turek, Paweł Lech, Natalia Dowgiałło‑Gornowicz

**Affiliations:** Department of General, Minimally Invasive and Elderly Surgery, Collegium Medicum, University of Warmia and Mazury, Olsztyn, Poland; Probios Ltd., Olsztyn, Poland

**Keywords:** bariatric surgery, constipation, nutrition, probiotics, sleeve gastrectomy

## Abstract

**INTRODUCTION:**

Sleeve gastrectomy (SG) is a safe metabolic and bariatric surgery but it often leads to postoperative gastrointestinal issues, such as constipation and bloating. The use of probiotics after SG seems to alleviate these symptoms.

**AIM:**

The aim of this study was to assess the impact of probiotic supplementation on macro- and micronutrient levels, specifically glycated hemoglobin (HbA1c), 3 months after SG. The secondary objective was to analyze the effect of probiotics on gastrointestinal symptoms.

**MATERIALS AND METHODS:**

This was a prospective, placebo-controlled study which included patients undergoing SG at a single center in Poland. Before the surgery, the patients did not note gastrointestinal diseases or symptoms. They were randomly assigned to the probiotics group (PG) or the control group (CG). Gastrointestinal symptoms and laboratory test results were assessed before surgery and 3 months postoperatively.

**RESULTS:**

A total of 24 patients were included in the study, of which 11 were assigned to the PG and 13, to the CG. Three months after surgery, the patients in the PG had slightly lower levels of HbA_1c_ than the CG participants (*P* = 0.01). The patients in the PG reported less constipation, better feeling of bowel movement completeness, and greater ease of defecation (*P* = 0.03; *P* = 0.01; *P* = 0.01, respectively) No differences in weight loss were found between the groups.

**CONCLUSIONS:**

Probiotics may lower HbA1c levels, effectively reduce constipation, contribute to a better feeling of bowel movement, and facilitate defecation after SG.

## INTRODUCTION

The World Health Organization defines obesity as an abnormal or excessive fat accumulation that presents a health risk.^1^ Worldwide statistics show that more than 16% of adolescents live with obesity and this number is constantly increasing. In Poland, 18.5% of people over 15 years are obese.^2^ Obesity has truly become a growing public health concern which needs multidisciplinary approach.

Metabolic and bariatric surgery (MBS) appears to be the most effective treatment in terms of weight loss and long-term improvement or remission of obesity-related diseases. Moreover, due to the expanded eligibility criteria for MBS, an increasing number of patients will be seeking surgical treatment.^3^ The most commonly performed bariatric procedure is sleeve gastrectomy (SG).^4^**^,^**^5^ Despite a low risk of complications and satisfactory outcomes, SG is associated with postoperative gastrointestinal complications, such as malabsorption of micro- and macronutrients, constipation, diarrhea, dysphagia, and abdominal pain.^6^**^,^**^7^ Over 50% of patients undergoing SG experience constipation, which makes it a significant problem.^8^**^,^**^9^ In 2023, 7450 SGs were performed in Poland, accounting for 82% of all MBSs.^10^ This sheds some light on how many patients may be affected by malabsorption of micro- and macronutrients, constipation, diarrhea, dysphagia, and abdominal pain.

Obesity is associated with reduced gut microbiota (GM) diversity and a high rate of micronutrient deficiency.^11^ After MBS, GM diversity and structure change even more, making probiotics increasingly relevant in this context, which is one of the reasons why the probiotics market is booming. The use of probiotics in patients undergoing MBS has been suggested to have a beneficial effect on weight loss, food intake, and vitamin levels.**^1^**^12^

Probiotics are beneficial live microorganisms that, when administered in appropriate doses, can modulate the GM structure and alleviate the effects of obesity, type 2 diabetes mellitus (T2DM), and other metabolic diseases. They typically include bacteria from the *Lactobacillaceae* and *Bifidobacteriaceae* families.^13^

Recent studies show that the modulation of GM composition, regulation of gut microbial metabolites, and improvement of intestinal barrier function are 3 major mechanisms through which probiotics regulate metabolic diseases.^14^^,^^15^ These mechanisms are closely related to the way gut bacteria ferment dietary fiber into short-chain fatty acids (SCFAs), which stimulate the release of incretin hormones (*GLP-1* and *PYY*) and fat cells to release leptin, thus improving glucose metabolism and insulin sensitivity, reducing appetite, and slowing gastrointestinal peristalsis.^15^^,^^16^^,^^17^ Probiotic treatment can also reduce homeostasis model assessment of insulin resistance, glycated hemoglobin (HbA_1c_), fasting blood glucose, and insulin resistance levels in patients with T2DM.^18^

Despite a growing number of studies on probiotics, a lack of solid clinical data and understanding of their mechanisms, compounded by short intervention periods, variability in strains, doses, and delivery methods, and the influence of external factors, such as medication, diet, and ethnicity, continues to hinder clear conclusions about their role in glucose metabolism regulation.^19^^,^^20^^,^^21^

SG is considered an effective bariatric technique for treating obesity, and probiotic supplementation may provide additional benefits, such as improving patients’ vitamin and metabolic profiles and mitigating many of the negative outcomes of surgical interventions.^22^**^,^**^23^ Administration of probiotics is simple, and it can contribute to enhanced postoperative comfort and recovery.^24^

## AIM

The aim of this study was to assess the impact of probiotic supplementation on macro- and micronutrient levels, specifically HbA_1c_, 3 months after SG. The secondary objective of the study was the analysis of the effect of probiotics on gastrointestinal symptoms postoperatively.

## MATERIALS AND METHODS

### Patients

This was an initial, prospective, triple-blind placebo-controlled study involving patients undergoing SG at a single center in 2024. It was a continuation of a previous study conducted by the same team.^24^ The postoperative consulting surgeon, the laboratory staff, and the patients were unaware of whether they received probiotics or placebo. The study was unblinded after 3 months, once all patients had completed the follow-up.

To be included in the study, the patients had to be over 18 years old, meet the eligibility criteria for MBS, and present no specific symptoms of gastrointestinal diseases. Exclusion criteria comprised gastrointestinal diseases, use of prokinetics or proton pump inhibitors (PPIs) 4 weeks before the surgery, and intake of antibiotics within at least 4 weeks before the study. Patients who refused to participate were also excluded.

### Data collection and outcomes

Data were collected before the surgery and 3 months postoperatively during in-person visits. The collected information included demographic information (age, sex, weight, height, waist circumference, presence of obesity-related diseases, and medication) and clinical data (laboratory test results and gastrointestinal symptoms). Laboratory tests involved complete blood count and ferritin, albumin, protein, HbA_1c_, total cholesterol, triglycerides, and vitamin B_12_ levels.

MBS outcomes were evaluated using standardized reporting, including percentage of total weight loss (%TWL) and percentage of excess weight loss.^25^ Gastrointestinal symptoms were assessed using a questionnaire before and 3 months after SG. The survey consisted of subjective assessment of frequency of bowel movements per week, occurrence of constipation, ease of defecation, and completeness of bowel movements. The last 2 questions were evaluated using a five-point Likert scale (1 corresponded to significant difficulty or incomplete evacuation, while 5 meant no difficulty or complete evacuation).

### Allocation

The participants were allocated in a random blinded manner and divided into 2 groups: the probiotics group (PG) and the control group (CG). The probiotic and placebo packages were provided by Probios Ltd. (Olsztyn, Poland) in identical packaging labeled with random numbers.

### Surgical technique and treatment

The surgeries were performed in accordance with the established guidelines.^26^ SG was performed using a 36- French bougie, starting 4–6 cm from the pylorus. The probiotic and placebo were both administered in disposable sachets to be dissolved in water. The probiotics used were unique strains from the Probios Ltd. collection, including *Lactobacillus plantarum* AMT14 (5 × 10^8^ colony-forming units [cfu]/g), *Bifidobacterium animalis* AMT30 (1 × 10^10^ cfu/g), and *Bifidobacterium breve* AMT32 (1 × 10^10^ cfu/g). These strains were a modified version of the existing laBIFID (Probios Ltd., Olsztyn, Poland) product, referred to as laBIFID plus. They are deposited in the Polish Collection of Microorganisms at the Institute of Immunology and Experimental Therapy of Polish Academy of Sciences in Wrocław, and are protected by Polish, European, and American patents. The placebo sachets contained only starch, matching the probiotics in quantity and color. Both products were to be refrigerated and taken once a day with the first meal for 3 months. Throughout the observation period, all patients followed the same regimen, including additional medication: low-molecular-weight heparin (0.4 ml subcutaneously for 10 days), omeprazole (40 mg orally for 30 days), ursodeoxycholic acid (600 mg orally for 90 days), and the multivitamin Floradix (Salus, Bruckmühl, Germany; orally for the entire study period). The patients adhered to a diet which was monitored by a hospital dietitian, and were instructed to drink 1.5–2 liters of fluids daily.

**TABLE 1:** Baseline patient characteristics

Parameter		Probiotics group	Control group	P value
Sex, n	Men	8	13	0.1
	Women	3	0	
Age, y		43 (11)	44.1 (11.4)	0.82
BMI before surgery, kg/m²		43.4 (5.3)	40.1 (4.4)	0.1
Waist circumference before surgery, cm		128.6 (15)	121.8 (13.6)	0.26
Type 2 diabetes, n (%)		1 (9.1)	2 (15.4)	0.82
Hypertension, n (%)		4 (36.4)	5 (28.5)	0.75

Data are presented as mean (SD) unless indicated otherwise.

Abbreviations: BMI, body mass index

**TABLE 2:** Outcomes 3 months after surgery

Parameter	Probiotics group	Control group	P value
BMI after surgery, kg/m²	34.8 (4.5)	31.7 (4.3)	0.09
%TWL	24.8 (4.8)	27 (8.3)	0.45
%EWL	48.9 (11.9)	58.4 (18.8)	0.16
Waist circumference after surgery, cm	107.2 (16.2)	99.9 (12.5)	0.24
Change in waist circumference, cm	21.5 (5.5)	29.6 (27.4)	0.34

Data are presented as mean (SD).

Abbreviations: %EWL, percentage of excess weight loss; %TWL, percentage of total weight loss; others, see TABLE 1

### Statistical analysis

A descriptive statistical analysis was conducted. All data were analyzed using Statistica software 13.PL (Tibco Software Inc., Palo Alto, California, United States). The normal distribution was checked using the Shapiro–Wilk test. Numbers and percentages were used for categorical variables. For continuous variables, median (interquartile range [IQR]) and mean (SD) were employed. The *t* test or the Mann–Whitney test were applied for independent variables. For categorical variables, we used the χ² test. A *P* value below 0.05 was considered significant.

### Ethics

The data were anonymized. The study was conducted in accordance with the ethical standards of the 1964 Declaration of Helsinki and its subsequent amendments. The study was approved by the Bioethics Committee of the University of Warmia and Mazury in Poland (1/2024).

## RESULTS

A total of 31 patients were eligible for includion in the study. Five patients refused to participate in the follow-up, and 2 participants were excluded. The follow-up rate was 77.4%. Finally, 24 patients were analyzed, with 11 assigned to the PG and 13 placed in the CG. Patient characteristics in both groups were similar, as illustrated in Table 1. There were no adverse effects associated with the use of probiotics, and no complications were recorded.

There were no intergroup differences in weight loss 3 months after SG, as shown in Table 2. Mean (SD) %TWL was 24.8% (4.8%) and 27% (8.3%) in the PG and CG, respectively (P = 0.45).

Patient baseline laboratory parameters were similar, as outlined in Table 3. Three months after the surgery, the PG participants showed markedly lower HbA_1c_ levels than the CG patients (*P* = 0.01).

The patients’ subjective feelings regarding the functioning of their digestive system differed considerably between the PG and CG (Table 4). The PG participants passed more stools per week than the patients in the CG (*P* = 0.03). Only 1 patient (9%) in the PG, and 8 patients (61.5%) in the CG reported constipation (*P* = 0.03). The patients in the PG reported a feeling of easier defecation and greater bowel movement completeness, as compared with the CG participants (*P* = 0.01; *P* = 0.01, respectively).

## DISCUSSION

The study indicates that the use of probiotics may exert a beneficial influence on glycemic control, resulting in lower HbA_1c_ levels. Moreover, while probiotics do not notably affect weight loss, they substantially reduce gastrointestinal discomfort during the first postoperative 3 months.

While several studies have examined the effects of probiotics on MBS outcomes, most have focused on bypass procedures, particularly Roux-en-Y gastric bypass (RYGB).^22^**^,^**^23^ In contrast, our study adds valuable insight into the effects of probiotics following SG, the most frequently performed bariatric procedure worldwide.

Our study showed that HbA_1c_ levels were significantly lower in the PG than the CG 3 months after SG. Similar findings were reported by Melali et al,^23^ who observed a similar trend in their study following RYGB at both 3 and 6 months after surgery.

Additionally, a study by Ismael et al^27^ demonstrated that treatment with the probiotic Hafnia alvei HA4597 improved metabolic outcomes, including glycemia, reduced body weight gain, decreased food intake, and lower fat mass gain 1 year after surgery. These findings suggest that probiotics could contribute to short-term glycemic management in patients with obesity, emphasizing the potential of GM as a therapeutic target in obesity treatment.

**TABLE 3:** Laboratory test results before and after surgery

Parameter	Baseline		P value	After surgery		P value
	Probiotics group	Control group		Probiotics group	Control group	
Hemoglobin, g/dl	14.3 (1.5)	13.8 (0.7)	0.27	14.4 (1.7)	13.4 (1)	0.08
Ferritin, mg/dl	159.5 (149)	87.5 (82.2)	0.15	116.7 (92.8)	103 (62.9)	0.67
Albumin, kg/m²	44.8 (1.9)	44.3 (2.7)	0.59	44 (1.9)	42.8 (2.2)	0.15
Protein, %	7.6 (0.5)	7.5 (0.5)	0.4	7.2 (0.4)	7 (0.3)	0.25
Glycated hemoglobin, %	5.7 (0.3)	6 (0.9)	0.28	5.4 (0.3)	5.8 (0.4)	0.01
Total cholesterol, mg/dl	193.2 (42.3)	198.5 (27.3)	0.72	184.3 (31.2)	179 (29.3)	0.67
Triglycerides, mg/dl	152.9 (74.3)	111.7 (50.7)	0.12	131.7 (58.2)	109.6 (37.4)	0.27
Vitamin B₁₂, pg/ml	457.4 (166)	519.7 (236.8)	0.47	409.3 (122.9)	475.9 (202.8)	0.35

Data are presented as mean (SD).

SI conversion factors: to convert hemoglobin to g/l, multiply by 10; total cholesterol to mmol/l, by 0.0259; triglycerides to mmol/l, by 0.0113; and vitamin B_12_ to pmol/l, by 0.738.

**TABLE 4:** Self-reported gastrointestinal symptoms following surgery

Parameter	Probiotics group	Control group	P value
Number of stools passed per week	7 (5–7)	3 (3–4)	0.03
Occurrence of constipation, n (%)	1 (9)	8 (61.5)	0.03
Feeling of ease of defecation	5 (5–5)	4 (4–4)	0.01
Feeling of bowel movement completeness	5 (5–5)	4 (4–4)	0.01

Data are presented as median (interquartile range) unless indicated otherwise.

The last 2 parameters were assessed using a Likert scale.

Currently, there is a limited body of research on the impact of probiotic supplementation on HbA_1c_ levels following SG. However, existing evidence suggests that probiotic supplementation exerts a modest yet significant effect on reducing HbA_1c_ levels in individuals with T2DM and prediabetes.^28^**^,^**^29^

A meta-analysis conducted by Hejazi et al,^30^ which included 32 randomized controlled trials, demonstrated a considerable reduction in HbA_1c_ levels following probiotic supplementation. Probiotics may improve glucose metabolism and lower HbA_1c_ through 3 primary mechanisms: enhancing the production of SCFAs which suppress lipolysis and reduce circulating free fatty acids; stimulating the secretion of incretin hormones, such as *GLP-1* and *GLP-2* while reducing glucagon, thereby improving insulin sensitivity; and modulating inflammatory, immune, and oxidative pathways by downregulating proinflammatory cytokines, such as interleukin (IL)-1β, IL-6, tumor necrosis factor α, and nuclear factor Κ-light-chain-enhancer of activated B cells.^30^ Together, these effects contribute to better glycemic control in individuals with insulin resistance and T2DM.^30^

A study by Wang et al^31^ showed that probiotics may significantly reduce lipopolysaccharide levels, alleviate endoplasmic reticulum stress, and improve insulin sensitivity. Existing knowledge shows that HbA_1c_ reflects average glucose control over the past 2–3 months; however, it does not capture glucose fluctuations in the postoperative period. Thus, future research should explore the impact of probiotics on glycemic variability over longer intervention periods.^31^

Additionally, several studies^32^**^,^**^33^ have reported that MBS not only enhances glycemic control through postoperative weight loss, but also induces metabolic changes that may lead to the remission of T2DM. Caiazzo et al^33^ observed that anatomical modifications resulting from MBS contribute not only to weight reduction but also to metabolic improvements, thereby enhancing glycemic stability and alleviating cardiovascular and hepatic comorbidities.

Our findings align with multiple studies^12^**^,^**^23^**^,^**^34^ that highlight the beneficial effects of probiotics on gastrointestinal function, particularly in reducing postoperative gastrointestinal discomfort. While most research has primarily focused on RYGB, where gastrointestinal issues stem from anatomical changes in the digestive tract, our study further emphasizes the role of probiotics in alleviating such discomfort in a broader context.

Swierz et al^35^ found short-term improvements in gastrointestinal symptoms, although they did not translate into significant changes in weight loss or quality of life. Nevertheless, these improvements are important for patient recovery, as they contribute to a smoother postoperative experience and may reduce the likelihood of hospital readmissions. Similarly, Sherf-Dagan et al^36^ observed that while probiotics did not markedly affect hepatic or inflammatory markers, the reduction in gastrointestinal symptoms considerably improved patient well-being during the critical postoperative phase.

Furthermore, the reduction in gastrointestinal symptoms, such as constipation and defecation difficulties, observed in our study reinforces the potential of probiotics in effective management of postoperative discomfort, thereby enhancing patient comfort and promoting recovery.

Our study found no differences in weight loss between the PG and CG, aligning with prior research.^12^**^,^**^35^ A meta-analysis by Wang et al^12^ suggested that probiotics might improve lipid metabolism and reduce food intake, but these effects were not consistently observed across the studies, which suggests that the impact of probiotics on weight loss may depend on other factors, such as probiotic strain, supplementation duration, and patient characteristics.

Some studies, including those by Ramos et al^34^ and Kazzi et al,^37^ reported improvements in gastrointestinal symptoms and biochemical markers, such as triglycerides and liver enzymes, following probiotic use after SG and RYGB. Similarly, a systematic review indicated that probiotics may help reduce body weight and waist circumference and improve lipid and liver markers, though their effects on glycemic control and quality of life remain uncertain.^38^ These findings suggest that while probiotics may not significantly influence weight loss, they could play a role in improving gastrointestinal health and modulating key biochemical markers, which could have long-term benefits for metabolic health. Stefura et al^39^ suggested that the composition of preoperative gastrointestinal microbiota may predict weight loss outcomes after MBS. Patients with better responses exhibited higher levels of *Firmicutes* in the oral microbiota and *Tannerella* (*Bacteroidetes*) in the intestinal microbiota, whereas those with less favorable weight loss had increased *Deltaproteobacteria* (*Proteobacteria*) and *Bernesiellaceae* (*Bacteroidetes*) in their gut.

Short-term benefits of with *Lactobacillus* supplementation after RYGB were reported by Woodard et al,^40^ though only during the early postoperative period. Mokhtari et al^41^ found similar effects in the first 3 months postsurgery, which diminished without continued supplementation. Despite some positive findings, the overall evidence on probiotics and weight loss remains inconsistent, likely due to study heterogeneity, variations in probiotic protocols, and methodological limitations.^12^**^,^**^35^**^,^**^42^ Additionally, these studies failed to adequately explore the influence of confounding factors, such as diet and medication (eg, antibiotics and PPIs).

All the results described above highlight the need for further research to determine the optimal probiotic strains, dosages, and supplementation duration for different surgical contexts.

### Study limitations and future directions

Our study contributes to the expanding body of literature by elucidating the complexities involved in assessing long-term effects of probiotics supplementation following SG and underscoring the necessity for standardized research methodologies. As it is a preliminary study, key limitations include a small sample size, short intervention period, and insufficient evidence on long-term colonization of probiotics and their persistence in the human gut. In addition, the efficacy of probiotics may be modified by extrinsic factors, such as medication (eg, metformin) and dietary diversity, depending on the ethnicity of the study groups, among others.^20^**^,^**^21^ Notably, the small sample size and short follow-up limit the generalizability of our findings and may overlook long-term impacts on weight and metabolic outcomes.

Although significant improvements in gastrointestinal symptoms were observed, our study did not comprehensively assess other potential benefits or adverse effects, such as the influence on immune modulation, psychological well-being, or sustained metabolic health. Given the very small number of patients enrolled and randomized, the findings of this study should be interpreted with considerable caution. Nevertheless, as an initial investigation, it yielded promising results and highlighted a potential direction for future research which should address these gaps to provide a more comprehensive understanding of the role of probiotics in postoperative management.

## CONCLUSIONS

Our study provides valuable insights into the role of probiotics in patients undergoing SG. Probiotic supplementation appears to be a promising strategy to enhance outcomes in postbariatric surgery patients, particularly by alleviating gastrointestinal symptoms and improving key metabolic parameters. Our findings prove that probiotic use markedly influences macro- and micronutrient levels 3 months after SG, with a notable reduction in HbA_1c_ levels.

Given the safety and potential benefits of probiotics, integrating them into postoperative care protocols could be a valuable strategy for enhancing patient recovery, reducing postoperative discomfort, and improving overall well-being. The analyzed formula may offer clinicians a useful tool for patient stabilization following SG. However, our study represents a preliminary investigation and requires confirmation in larger, well-designed future studies.

## JOURNAL INFORMATION

Videosurgery and Other Miniinvasive Techniques is an official journal of the Videosurgery Foundation.
